# Computed tomography versus sleep endoscopy (DISE) to predict the effectiveness of mandibular advancement device in adult patients with obstructive sleep apnea: A systematic review

**DOI:** 10.1371/journal.pone.0327974

**Published:** 2025-07-10

**Authors:** Pedro Cebola, Cristina Caroça, Helena Donato, Sara Dias, João Paço, Cristina Manso

**Affiliations:** 1 Egas Moniz Center for Interdisciplinary Research; Egas Moniz School of Health & Science, Almada, Portugal; 2 Sleep Center, CUF Tejo Hospital, Lisboa, Portugal,; 3 CHRC, NOVA Medical School, Universidade Nova de Lisboa, Lisboa, Portugal; 4 Documentation and Scientific Information Service, Centro Hospitalar e Universitário de Coimbra Coimbra, Coimbra, Portugal; 5 EpiDoC Unit, Centro de Estudos de Doenças Crónicas da NOVA Medical School, Universidade Nova de Lisboa, CEDOC—Campus Sant’Ana, Pólo de Investigação, NMS, UNL, Edifício, Lisboa, Portugal; 6 EpiSaúde Sociedade Científica, Évora, Portugal; 7 Escola Superior de Saúde do Instituto Politécnico de Leiria, Unidade de Investigação em Saúde, Leiria, Portugal; Hospital São Lucas da PUCRS: Hospital Sao Lucas da PUCRS, BRAZIL

## Abstract

**Study objectives:**

Obstructive sleep apnea (OSA) has a high prevalence in the global population and is a significant sleep disorder. One of the non-invasive options to treat obstructive sleep apnea is the mandibular advancement device. 2 methods can help predict the success of mandibular advancement devices, and they are Neck computed tomography (CT) and Drug-induced Sleep endoscopy (DISE). The objective of this study was to determine which method is better at predicting the success of a mandibular advancement device in the treatment of obstructive sleep apnea.

**Methods:**

PubMed, Embase and Web of Science Core Collection databases were comprehensively searched. A total of 1809 was obtained through the extensive search of the three databases mentioned above (Embase = 952; PubMed = 508; Web of Science = 349). The exclusion criteria were studies lacking clear and replicable methodologies; research involving pediatric and adolescent participants, as well as individuals with craniofacial malformations; and Animal studies, conference abstracts, editorials, case reports, book chapters, and review articles.

**Results:**

Based on the inclusion criteria, mainly because of the lack of direct comparison between CT and DISE for the prediction of MAD success, two articles were selected for this systematic review. The conflicting findings between the two studies underscore the complexity of OSA management and challenges associated with predicting treatment outcomes using different diagnostic modalities.

**Conclusions:**

Although CT offers detailed anatomical information regarding airway morphology, DISE provides dynamic visualization of upper airway collapse during sleep, which may better simulate real-life conditions and MAD responses.

## Introduction

A sleep disorder that is highly prevalent in the global population is obstructive sleep apnea (OSA), and it is characterized by the collapse of the upper airway (UA), which can be partial or total, during sleep due to recurrent episodes of apnea or hypopnea [1]. Respiratory effort is associated with recovery from regular breathing after the aforementioned respiratory episodes, leading to daytime hypersomnolence, sleep fragmentation, and decreased sleep quality [2].

There is a wide variation in the epidemiology of OSA, ranging from 14% to 84% in men and 5% to 61% in women. Nonetheless, it is believed that these numbers underestimate the true prevalence, with many cases going undiagnosed [2–4].

International guidelines recommend diagnosing OSA through sleep studies such as polysomnography (PSG) or Home Sleep Apnea testing (HSAT) [2,5]. When there is a 90% reduction in airflow for at least 10 seconds, it is defined as apnea. In contrast, when there is at least a 50% reduction in airflow and a 3% reduction in oxygen saturation for at least 10 seconds, it is considered hypopnea. This latter respiratory event in adults can also be classified as an oxygen desaturation of 4% or more from the pre-event baseline in some accredited sleep centers. The apnea-hypopnea index (AHI) determines the severity of OSA: it is considered that there is no OSA when the AHI is below 5, mild when it is between 5–15, moderate when it is between 15–30, and severe when it is above 30. Asymptomatic individuals are only diagnosed with OSA if their AHI is > 15 [2,6].

Regarding the severity of OSA, some authors have found that relying solely on the AHI is insufficient, as it fails to capture the heterogeneity of the disease. To predict the long-term impact factors of OSA and given the absence of a validated scale, the international consensus suggests considering the following criteria: AHI, the duration of oxyhemoglobin desaturation below 90% as an indicator of hypoxemia; daytime sleepiness; the degree of obesity measured by body mass index (BMI); and the presence of comorbidities linked with OSA, such as hypertension (especially if it is resistant to treatment or exhibits a non-dipper pattern), type 2 diabetes mellitus, dyslipidemia, coronary artery disease, stroke, heart failure, or atrial fibrillation [7].

OSA is frequently associated with comorbidities, such as cardiovascular and metabolic diseases, and is observed in half of patients. One prevalent example is persistent secondary arterial hypertension and non-dipping nocturnal hypertension profiles. The prevalence of OSA is high in patients with type 1 diabetes mellitus. Additionally, OSA is linked to cerebrovascular diseases, with a prevalence of 62% in moderate OSA and 30% in severe OSA [2,8,9].

Continuous positive airway pressure (CPAP) has been the primary treatment for OSA since its publication in 1981. It is recommended for symptomatic OSA, patients with concurrent health conditions, or those diagnosed with moderate-to-severe disease through PSG [1]. CPAP therapy improves the quality of life and reduces the risk of accidents, cardiovascular events, and mortality associated with OSA. However, long-term adherence rates range from 30% to 50% [2,10,11].

As not all patients tolerate CPAP well, other treatment options are needed. Mandibular advancement devices (MAD) are the initial non-invasive option for non-adherence to CPAP, and the initial treatment approach applies to mild and moderate OSA cases that do not involve additional health conditions and lack significant oxygen level impacts. MADs position the mandible forward to manage upper airway openness during sleep, effectively reducing AHI. Although MADs may not achieve the same level of efficacy as CPAP in managing airway obstruction, they exhibit superior clinical and scientific adherence [2,11]. Despite their documented lower effectiveness compared to CPAP, MADs provide an alternative for patients who cannot tolerate CPAP therapy. An increasing number of articles indicate that using MADs for treating OSA is effective in improving PSG indices and objective and subjective measures of sleepiness, blood pressure, neuropsychological functioning, and quality of life. Research suggests that MADs reduce AHI by ≥50% in approximately 60–70% of patients, with AHI dropping below five events per hour in approximately 35–40% of cases [12].

These two complementary diagnostic tests, computed tomography (CT) and Drug-Induced Sleep Endoscopy (DISE), are not frequently used in OSA diagnosis and can be associated with significant expenses [13].

MADs can be categorized as either standardized (without the need for dental arch impressions) or customized (requiring impressions of the dental arches). Non-titratable MADs maintain the mandible in a fixed protruding position throughout the treatment without the ability to adjust it. In contrast, customized MADs are titratable, featuring mechanisms that allow incremental adjustments in mandibular protrusion based on the patient’s response to therapy. The increase in mandibular protrusion in titratable MADs is akin to the titration process used in CPAP therapy [14].

Factors related to anatomy and neuromuscular physiology are pivotal in the pathophysiology of OSA. Imaging diagnostic modalities can offer valuable insights into OSA evaluation and identify patients who are likely to benefit from MAD [2,15]. Among these methods, CT provides detailed images of the upper airway bone and soft tissues from the nasopharynx to the larynx. Axial plane images with high resolution (1–2 mm thickness) enable accurate measurements of the length and cross-sectional dimensions [2,16].

Although CT provides a fixed, planar depiction of complex, dynamic anatomical structures of the head and neck, it is considered a valuable predictive tool. Substantial variation in these measurements has been observed between asymptomatic individuals and those diagnosed with OSA. Moreover, cephalometry has been utilized to evaluate how changes in body position affect upper airway anatomy and function in individuals with OSA. Morphological predictors, such as retrognathia, micrognathia, long face, inferior hyoid bone positioning, accentuated mandibular plane, upper airway, soft palate, and tongue narrowing, have been identified. CT-based cephalometric analysis aids in the diagnosis and preoperative assessment of surgical OSA therapy [2,17].

Various surgical procedures and positional therapies have been introduced to enhance therapy adherence and efficacy when used alone or in combination [18]. Personalized treatment is crucial to achieve optimal long-term outcomes. To this end, tools such as DISE have been presented. Since its introduction in 1991, DISE has enabled the real-time evaluation of areas prone to vibration and collapse using flexible nasopharyngoscopy under sedation [2,19].

Research indicates that approximately 50% of surgical strategies are adjusted after DISE, in contrast to evaluations conducted while the patients are awake. Certain DISE observations correlate with the therapeutic approach outcomes [2,18]. However, DISE has indications and contraindications. Absolute contraindications included ASA 4 status, pregnancy, and allergies to DISE sedatives. Severe obesity is generally considered a relative contraindication, although specific UA features may warrant DISE consideration, even in these cases [2,18].

To enhance the accuracy of predicting MAD therapeutic success, adjustable intraoral devices and techniques such as chin lift and jaw thrust (*Esmarch*) can be used during DISE. These interventions should be carefully assessed to minimize patient discomfort and the associated micro-arousals [2,19].

This systematic review aimed to determine the most effective method for predicting the efficacy of MADs in OSA therapy. To date, no systematic review has directly compared the predictive values of CT and DISE for MAD therapy outcomes in patients with OSA. By addressing this gap, our study provides original insights into the diagnostic workup and individualized treatment planning for non-CPAP therapies. Given the imperative to individualize treatment and ensure safety in MAD prescriptions, this topic is of paramount importance.

## Materials and methods

### Protocol register and ethics

This systematic review followed the PRISMA-P (Preferred Reporting Items for Systematic Reviews and Meta-Analyses Protocols) guidelines [2,20]. Furthermore, the protocol of this systematic review was registered with the PROSPERO database on November 2, 2021 (registration number: CRD42021282845), and was published previously in 2023 [2].

As this systematic review did not involve recruiting patients or gathering personal information, ethics committee approval was unnecessary.

### Research question

Which complementary diagnostic method is more effective in evaluating and predicting the success of MAD treatment in adult patients with mild-to-severe OSA (AHI greater than 5/h): CT combined with cephalometry or DISE using propofol as a sedative and a system to assess UA obstruction?

#### Review question.

Adhering to the PICO framework, the research question is structured as follows.

Patient: Adult patients from diverse ethnic backgrounds and genders undergoing MAD therapy for OSA;

Intervention: OSA patients evaluated using either DISE or CT with cephalometry to assess UA obstructions;

Comparison: OSA patients evaluated using either DISE or CT with cephalometry to predict MAD treatment outcomes;

Outcome: To assess whether UA obstructions identified through CT with cephalometry more accurately predict the success of MAD treatment than DISE.

### Eligibility criteria

#### Types of studies.

We included randomized clinical trials, non-randomized prospective or retrospective clinical studies, case-control studies, cohort studies, and case series. Given the scarcity of available research on this topic, we decided to broaden the scope of our research.

#### Patients.

The study included adult participants aged 18–65 years diagnosed with OSA (AHI > 5/h) using PSG, following recognized diagnostic criteria, with no restrictions based on gender or ethnicity.

#### Intervention.

One group underwent DISE with propofol as a sedative and utilized a system for classifying UA obstructions to predict the effectiveness of MAD treatment, whereas the control group underwent neck CT scans along with different cephalometric measurements to assess MAD treatment outcomes.

#### Outcome indicators.

(1) **Primary outcomes:** The main measurements included craniofacial characteristics, cephalometric assessments, and the location and type of upper airway obstruction.
(2) **Secondary outcomes:** Additional metrics included mean values of AHI (e.g., > 50% improvement and reductions to < 15/h or < 5/h), average SaO2, time spent under 90% SaO2 (T90), improvements in Epworth Sleepiness Scale (ESS) and/or Pittsburgh Sleep Quality Index (PSQI), as assessed in the initial and follow-up PSG, and heart rate, SaO2 levels, and bispectral index (BIS) scores recorded during DISE.

#### Exclusion criteria.

The criteria for exclusion, as determined by our team, encompass: (1) Studies lacking clear and replicable methodologies; (2) Research involving pediatric and adolescent participants, as well as individuals with craniofacial malformations; and (3) Animal studies, conference abstracts, editorials, case reports, book chapters, and review articles

### Information sources

We employed a comprehensive search strategy focusing on subjects and topics of interest conducted on February 18, 2024. The databases searched included PubMed, Embase, and Web of Science Core Collection. Additionally, manual searches were conducted using reference lists and citations of full-text articles eligible for inclusion in this systematic review.

### Search strategy

We conducted a systematic search of the PubMed, Embase, and Web of Science databases. Additionally, we manually searched the references and citation lists of the eligible full-text articles included in this systematic review.

### The key search terms employed were as follows

- “obstructive sleep apnea” – “obstructive sleep apnea syndrome,” “sleep apnea syndrome,” “snoring,” ”sleep-related breathing disorder*”, ”sleep respiratory disorder*”, “sleep-disordered breathing,” “OSA.”
- Prediction – “predict*”; “prognostic*” prognostic *
- anatomy-base outcome - “anatomic obstruction,” “computed tomography”; “CT”; “Drug-induced sleep endoscopy”; “DISE”; “sleep endoscopy”; “cephalometry”; “cephalometric”;
- Oral appliances - “mandibular advancement device”, “””mandibular advancement appliance”,” “mandibular advancement splint”, “””mandibular repositioning device”,” “mandibular repositioning appliance”, “””mandibular repositioning splint”,” “oral appliance”, “””oral device”,” “dental appliance”, “””dental device.

Boolean operators (AND/OR) were used to merge searches. Only studies published after 1990 and written in English were included, as the DISE began in 1991 [17]. English language inclusion aims to encompass a representative selection of widely recognized studies in the scientific community.

#### Data filtering and extraction.

***Eligible studies were selected in two phases.*** During the initial phase, two reviewers (PC and CC) evaluated the titles and abstracts of the studies. Inclusion criteria were as follows: (1) adults diagnosed with OSA (AHI > 5/h) via polysomnography (PSG) and treated with MAD, who underwent both CT with cephalometry and DISE with propofol sedation, using a system to classify upper airway obstruction before MAD initiation; (2) assessment of treatment outcomes through subsequent PSG recordings; and (3) studies evaluating UA obstruction with CT, cephalometry, and/or DISE in OSA patients undergoing MAD treatment or predicting outcomes of MAD therapy in OSA. The included studies were published after 1990 and were written in English. The exclusion criteria were (1) studies without specified treatment modalities or using therapies other than MAD; (2) absence of OSA (AHI < 5/h); (3) editorials, reviews, conference abstracts, case reports, and book chapters; (4) patients under 18 or over 65 years of age; (5) unclear inclusion and exclusion criteria; and (6) studies lacking clear and reproducible methodologies.

In the second phase, the full texts of potentially eligible studies from the initial phase were independently reviewed by both reviewers. Studies that did not meet these criteria were also excluded. Discrepancies between the reviewers were resolved through discussion with a third reviewer to achieve a consensus.

Data extraction was conducted by one reviewer and verified by another reviewer.

We synthesized the findings from the included studies narratively and manually screened references of studies identified in the second phase to ensure comprehensiveness.

#### Literature quality (bias) assessment.

The methodological rigor of the studies was evaluated using the QUADAS-2 tool to assess both the bias risk and applicability in primary diagnostic accuracy studies. This tool consists of four main domains: patient selection, index testing, reference standards, and flow and timing.

Each domain was scrutinized for risk of bias, with additional consideration given to applicability concerns for patient selection, index test, and reference standard. Signal questions were incorporated to aid bias assessment. The application of QUADAS-2 involved four phases: 1 – defining the review question; 2 – customizing the tool for the review and creating review-specific guidelines; 3 – constructing a flowchart for the primary studies; 4 – assessing bias risk and applicability concerns.

## Results and discussion

### Literature search

**Fig 1:**
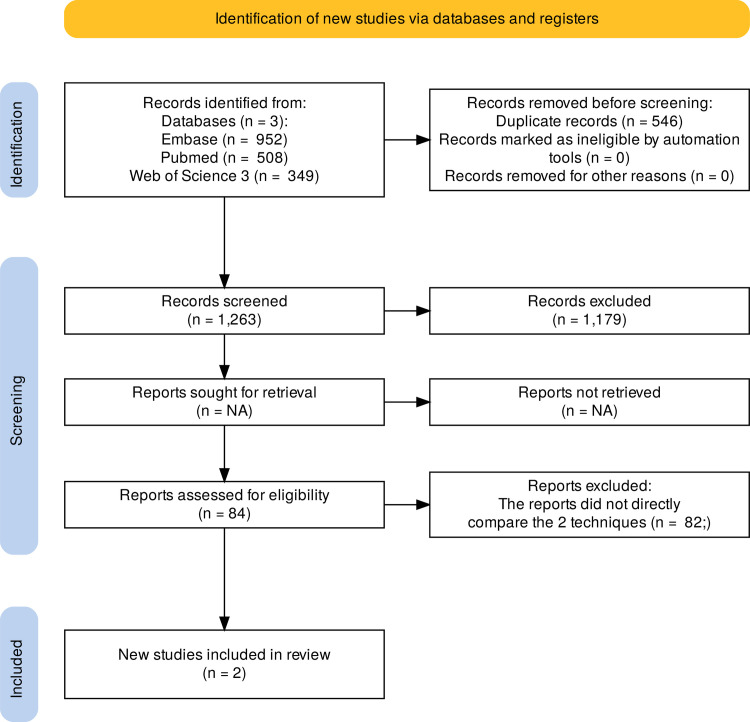
PRISMA flowchart.

A total of one thousand eight hundred and nine references (n = 1809) were obtained through the extensive search of the 3 databases mentioned above (Embase = 952; PubMed = 508; Web of Science = 349). A total of 546 references were deleted at the start because they were duplicated. Of the 1263 references that remained after the removal of duplicates, 1179 were excluded after reviewing the titles and abstracts because they were not appropriate for this systematic review. The reasons remained: sampling with children or adolescents, Sampling with animals, therapeutic approaches that were not MAD, case reports and reviews, and patients without OSA. The full texts of the remaining 84 references were retrieved. Based on the inclusion criteria, mainly because of the lack of direct comparison between CT and DISE for the prediction of MAD success, 2 articles were selected for this systematic review (Fig 1).

### Overview of study design and diagnosis of OSA

Both studies utilized a prospective design with a single evaluation point to measure treatment efficacy [15–21]. The duration of the studies was specified in two papers, which lasted either 2 weeks [21] or 3 months [15]. The number of subjects varied significantly, from 30 [21] to 72 [15], with an average of 51 individuals per trial.

Two studies used full-night polysomnography to diagnose OSA in patients [15–21]. Saleh et al. used the SOMNOscreen™ plus system (SOMNOmedics, Germany) [21]. The diagnostic cutoffs were set at the same thresholds; both studies recruited patients with an AHI > 5/h. Van den Bossche et al. used the American Academy of Sleep Medicine (AASM) 2007 classification score for obstructive sleep apnea [15], while Saleh et al. used the International Classification of Sleep Disorders-3 (ICSD-3) diagnostic criteria. Although a specific version of the AASM scoring manual has not been reported, it is presumed that the standard AASM criteria were used for respiratory event scoring [21]. All study participants were grouped based on OSA severity. Aligned with the clinical research question of this review, each study examined the link between treatment results derived from PSG data and anatomical factors measured through cephalometry and DISE [15–21].

### Craniofacial features, cephalometric evaluations, and location and type of UA obstruction

#### DISE.

Two articles used DISE to assess the UA and its collapse by introducing flexible nasofibroscopy through the nose [15–21]. Saleh et al used a propofol dosage of 1 mg/kg, followed by 20 mg doses every 2 minutes until the onset of the snoring-apnea cycle. The target-controlled infusion of propofol used was 1.5 μg/mL [21]. Van den Bossche et al. did not solely use propofol; instead, it began with endovenous administration of midazolam (1.5 mg), followed by propofol administered via target-controlled infusion (2.0–3.0 μg/ml) follow [15].

Both categorized the collapses of the UA in different ways: the location of the obstruction (soft palate, tonsils, tonsil base, and epiglottis); the mechanism of the obstruction (anteriorposterior, concentric, and posterior-lateral); the intensity of the obstruction (vibration, collapse, and collapse and vibration); and the duration of obstruction in the respiratory cycle (inhalation and exhalation) are classified [21]. According to Van den Bossche et al., the location of obstruction (palate, oropharynx, tongue base, pharyngeal lateral walls, and epiglottis), degree of collapse (complete, partial, or none), and direction of collapse (anteroposterior, laterolateral, or concentric) were classified [15].

The patients were divided into three groups: “complete response” (AHI < 5/h), “partial response,” and “no response” when the MAD was used to treat OSA, in Saleh et al. article. They found that the complete responders did not exhibit epiglottis collapse in the DISE (n = 6), and that they differed significantly from the partial responders (p = 0.029) [21].

In Van den Bossche et al. article, the patients were divided into four groups: “response,” “no response,” “deterioration,” and “no deterioration” when the MAD was used to treat OSA. The term ‘responses’ refers to a 50% decrease in the AHI, while ‘deterioration’ is primarily defined as an AHI increase of more than 10% from the baseline measurement. During the assessment, a notable contrast emerged between the individuals who showed a positive response and those who did not. Specifically, there was a marked variation in the occurrence of tongue base collapse, with a higher prevalence observed among those who responded positively than among those who did not (statistical significance, p = 0.0404). Additionally, in terms of palatal collapse, the disparity between responders and non-responders was evident, with a greater incidence noted among non-responders (statistical significance, p-value = 0.0240). At the initial assessment, notable differences were observed between patients who experienced deterioration and those who did not. Specifically, there were significant variances in baseline characteristics related to certain DISE parameters. These differences were particularly evident in the complete concentric palatal collapse (CCCp) measurement (statistical significance, p-value = 0.0494) and the occurrence of complete laterolateral oropharyngeal collapse (statistical significance, p-value = 0.0364) [15] (Table 1).

**Table 1:** PICO—Computed tomography versus sleep endoscopy to predict the effectiveness of mandibular advancement devices in adult patients with obstructive sleep apnea.

	Patient	Intervention	Comparison	Outcome
*Saleh,Y* (2022)	30 patients with OSA	MAD Treatment applied after evaluation of DISE and CBCT	PSG and CBCT after	DISE and CBCT can be useful tools for assessing the success of MAD in the treatment of OSA
*Bossche, K* (2022)	72 patients with OSA	MAD Treatment applied after evaluation of DISE, awake nasendoscopy and CBCT	PSG: reduction in the apnea–hypopnea index (AHI) of ≥50% and deterioration as an increase of ≥10% during MAD treatment	DISE to be the most robust examination associated with MAD treatment outcome, with tongue base collapse as a predictor for successful MAD treatment and CCCp as an adverse DISE phenotype

AHI – Apnea-hypopnea Index; MAD – Mandibular advancement device; CT – Computed Tomography; DISE – Drug-induced sleep endoscopy; UA – Upper airway; SBD – Sleep breathing disorder; BZD – benzodiazepine; ATD – antidepressant; OR – Odds Ratio; CCCp’s – complete concentric palatal collapse

According to the odds ratios (OR), the two largest effects observed were the collapse of the responders’ tongue base (OR 3.29, 95% CI 1.02–10.64; p = 0.0464) and CCCp degradation (OR 28.88, 95% CI 1.18–704.35) [15]. (Table 1). Parte inferior do formulário

#### Computed tomography.

Regarding computed tomography, each of the two articles examined the potential collapse of the UA differently [15–21]. According to Saleh et al., the patients underwent Cone-Beam Computerized Tomography (CBCT) and were categorized as “more than 110 mm^2^” is the normal airway cross-sectional area; “52–110 mm^2^” is the mild to moderate range, and “less than 52 mm^2^” is the severe range [21]. In contrast, Van den Bossche et al. used computational fluid dynamics (CFD) to perform awake baseline low-radiation-dose CT scanning. From these data, the effective UA volume, excluding mouth leakage, was calculated. Measurements were taken for the velopharynx, oropharynx, hypopharynx, and distinct regions of the pharynx, as well as for the overall pharyngeal volume. Additional anatomical parameters, such as the minimal cross-sectional area and UA resistance were also assessed [15].

Patients were examined using CBCT both before and after receiving MAD. They showed a significant improvement in the following airway parameters in the patients after receiving MAD treatment: total volume of air (P < 0.001) and minimum and maximum cross-sectional areas (p = 0.047 and 0.038, respectively) [21] (Table 1).

In contrast, Van den Bossche et al. found no significant differences in UA CT parameters between patients who responded to the MAD and those who did not. Additionally, they suggested that there were no significant differences in the UA parameters on CT between patients who were deteriorating and those who were not [15] (Table 1).

#### DISE versus computed tomography.

The authors of the two articles included in this systematic review provided differing conclusions regarding the differences between CT and DISE [15–21]. According to Saleh et al., both DISE and CT can be valuable tools for predicting and analyzing the effectiveness of MAD treatment for OSA [21]. In contrast, Van den Bossche et al. stated that DISE is a more thorough examination for analyzing the likelihood of MAD success in the treatment of OSA [15] (Table 1).

#### Heart rate and BIS.

None of the articles addressed any of these variables during DISE [15–21].

#### Mean values of AHI.

The primary outcome variable was AHI in 2 studies [15–21]. The average AHI values were used to summarize the outcomes in the study by Saleh et al. article [21]. However, Van den Bossche et al. reported median AHI values [15].

Both studies showed a significant decline in AHI when the MAD was used [15–21], and one study found that the mean AHI was 31.4 ± 15.6/h before the MAD and 13.8 ± 15.2/h (P = 0.001) after MAD [21]. Conversely, in the study by Van den Bossche et al., the AHI’s median (interquartile range [IQR]) improved from 15.6 (10.4–23.5) to 9.0 (4.3–16.0) events/h (p < 0.0001) [15] (Table 1).

#### Average SaO_2_.

Concerning this result, only Van den Bossche et al. have references on the Average SaO2. This remained unchanged both before and after the use of MAD, as seen in the following example: initial = 95.3% (94.1%–96.1%) and final = 95.3% (94.2%–96.0%) [15].

#### Time Under 90% SaO_2_ (T90).

None of the studies included in our systematic review used the Time Under 90% SaO2 (T90) variable [15–21].

#### Enhancement in ESS scores and/or in PSQI scores.

This secondary outcome, measured using a questionnaire, was found in both studies [15–21]. However, only ESS, which analyzes excessive daytime sleepiness, was used in both cases [15–21]. In the study by Saleh et al., the initial median score of the ESS was 11.4 ± 5.3; however, the value after applying the MAD was not mentioned. Mention just that The ESS score significantly improved (P = 0.004) for the participants in the study following their adaptation to MAD [21]. In Van den Bossche et al., study, the use of the MAD resulted in a fall in the ESS from a median (IQR) score of 9/24 (5–12) to 6/24 (3–10) (p < 0.0001) [15].

After using the MAD, patients’ sleep quality improved according to Saleh et al., who used the PSQI. Specifically, prior to receiving MAD, the patient’s mean PSQI value was 17.5 ± 3.1; however, following therapy, it dropped to 6.5 ± 3.6 (p = 0.001) [21].

### Literature quality (bias) assessment (QUADAS-2)

#### Risk of bias.

**Fig 2:**
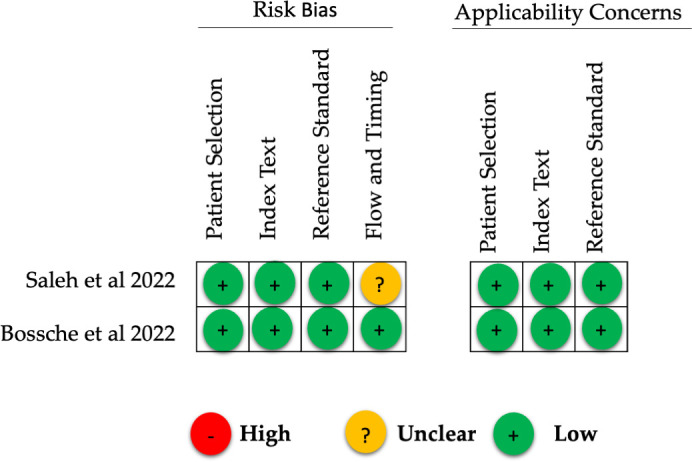
Results of QUADAS-2 assessment.

Fig 2 presents the assessment outcomes of study quality for both the included studies. We considered Van den Bossche et al. to be at low risk across all four risks of bias domains: patient selection, index test, reference standard, and flow and timing [15]. Two studies were at low risk of bias for three domains (patient selection, index test, and reference standard), but at unclear risk of flow and timing [21].

The included studies enrolled consecutive patients [15–21]. Finally, it is unclear in Saleh et al. ’s study whether there was a suitable time gap between the index test and the reference standard [21].

#### Applicability concerns.

There were no applicability concerns for the two studies across the three applicability domains of patient selection, index test, and reference standard [15–21].

## Discussion

Our study synthesizes current research findings on the diagnostic efficacy of neck CT and DISE in predicting the therapeutic success of MAD for managing OSA. This review involved a rigorous and transparent systematic literature search. Of the 1809 references searched in Embase, PubMed, and Web of Science, only two analyzed and compared CT and DISE in predicting MAD success for OSA. This demonstrates that this is an underexplored topic and that the study that our research group will produce shortly, taking into account all of the knowledge absorbed through the systematic review, will be relevant and lead to scientific advancements.

Consensus is lacking regarding the standards for the success of MAD therapy; nonetheless, recent scientific evidence indicates that factors such as low body mass index (BMI), supine-dominant OSA, neck circumference less than 40 cm, and sex may impact treatment outcomes with MAD [19].

The effectiveness of MAD in patients with OSA may differ based on the severity of the condition. Variables such as enlarged palatine tonsils or prominent pharyngeal pillars could contribute to less favorable outcomes, potentially resulting from partial collapse or compression of the base of the tongue. Given these considerations, targeting interventions that focus on the anterior positioning of the soft palate or base of the tongue before treatment could enhance the efficacy of MAD therapy [22]. Regardless of the location of UA obstruction, it is crucial to remember that multiple-level obstruction is the most common level of obstruction, whether assessed by CT or DISE [21].

Recent advancements in CT technology have enabled volumetric measurements using specialized software such as Sicat Air ® [17], generating three-dimensional images of craniofacial structures, mandible, hyoid bone, spine, and airways. Another aspect explored for its potential to predict MAD success through CT evaluation included assessing the angle of the skull base and measuring the distance between the sella turcica and the deepest point in the posterior cranial fossa [22]. MAD can also be predicted by factors such as total air volume and minimum and maximum cross-sectional area [21]. However, Bossche et al. (2022) stated that isolated CT analysis is not a reliable predictor of MAD or deterioration [15].

While CT scans are beneficial in specific scenarios, their routine use is not recommended because of associated expenses, restricted availability, and comparatively high radiation exposure [2,12,23].

An additional parameter integrated into DISE evaluates and strategizes the optimal therapeutic intervention for OSA [2,24–25]. This parameter involves personalized bite registration conducted by a sleep dentist before DISE to determine the patient’s Maximum Comfortable Protrusive Position (MCP). Consequently, the predictive value of DISE, combined with MCP bite registration, was assessed for MAD treatment outcome [26]. It is suggested that patients showing confident responses to MAD often exhibit a substantial increase in complete UA volume, indicating that MAD efficacy correlates with UA volume augmentation. Conversely, the absence of velopharyngeal volume increment appears to be associated with treatment deterioration [2,15,27].

Additionally, evidence suggests a link between the response to MAD treatment and the overall volume of the UA, especially focusing on the velopharyngeal region [14,28–29]. Another anatomical predictor that was evaluated was epiglottic collapse. When there is no collapse, the MAD have a higher chance of success [21].

Tailoring diagnostic and treatment methods for OSA is crucial for enhancing patient outcomes. Adopting personalized medicine for OSA represents a novel strategy, as traditional methods still need to adequately address the critical diagnostic and therapeutic aspects of the condition. A personalized therapeutic approach aims to meet each patient’s specific needs, thereby ensuring appropriate and optimal treatment.

One of the key clinical takeaways of this review is the importance of aligning diagnostic tools with patient-specific characteristics to optimize MAD therapy outcomes. DISE stands out for its ability to simulate sleep-related airway behavior, providing functional information that can directly inform treatment decisions. Identifying unfavorable collapse patterns—particularly tongue base collapse and complete concentric palatal collapse (CCCp)—can help clinicians avoid prescribing MADs to patients unlikely to respond [15,21,26]. This selective approach not only increases treatment efficiency, but also minimizes unnecessary costs and delays in care.

In contrast, CT offers a static but valuable anatomical overview that may be especially useful in treatment planning when craniofacial morphology plays a central role or when DISE is unavailable. Its ability to quantify airway volume and assess skeletal structures can support clinical decisions in a complementary way, though it lacks the dynamic insight of DISE [13,17,23].

From a practical standpoint, these modalities should not be considered as mutually exclusive. Rather, combining anatomical and functional assessments may represent the most effective path toward personalized therapy in OSA, particularly in cases where CPAP intolerance necessitates a tailored, non-invasive alternative, such as MAD.

Although there have been considerable improvements in identifying and treating this condition in recent years, implementing a tailored and holistic approach promises to enhance the management of OSA patients [2,30].

Both Neck CT and DISE allow three-dimensional evaluation of the UA. While neck CT is performed with the patient conscious and DISE requires sleep induced by medication, there is some convergence between the two methods [31]. CT imaging provides insight into obstructions related to the lateral walls of the oropharynx, as demonstrated by Zhang et al. in 2014. This suggests a potential partial replacement for DISE, given its requirements for a trained team, specialized facilities, time, expense, and the need for pharmacological sleep induction [2,30].

Although relatively few studies have directly compared DISE with Neck CT, both examinations are valuable for evaluating patients with OSA, each providing unique information [30]. As discussed earlier, DISE offers dynamic insights, whereas Neck CT provides more static information [2,18].

Studies comparing CT and DISE yield different results based on our knowledge and research [15,21]. According to one study, there are no significant differences or benefits of using DISE instead of CT for predicting MAD success [21]. In addition, Bossche et al. (2022), DISE is the only test that can predict the success of MAD for treating OSA [15].

However, the conformation of the UA, as assessed using DISE and/or Neck CT, fails to fully capture the intricate pathophysiology of OSA. This condition is multifaceted and cannot exclusively be attributed to anatomical factors. Variables such as arousal threshold, respiratory control stability, and genioglossus muscle responsiveness play crucial roles in OSA and must be considered as they can significantly differ between sleeping and awake states [2,32]. Some articles suggest that a potentially less invasive method for predicting treatment success and identifying patients responsive to MADs is through standard or temporary MADs. These possibilities are often more cost-effective and frequently demonstrate efficacy in managing OSA [2,33].

It is also important to note that while we can predict who will respond best to a MAD, it is equally crucial to understand adherence. However, success cannot be achieved without good treatment adherence. Therefore, several factors beyond anatomical considerations are important for analysis. Factors that may positively impact adherence to MAD include good communication skills of the practitioner, the use of a custom-made MAD, effective therapy, early detection of adverse effects, gradual adjustment of the MAD dosage, and favorable initial experience with the MAD. Conversely, elements that might deter commitment to MAD therapy include experiencing side effects such as discomfort or dental pain, opting for a thermoplastic MAD instead of a custom-made device, personality traits such as type D personality, MAD efficacy, dental procedures during MAD therapy, and an unsatisfactory initial experience due to inadequate guidance from healthcare providers. The literature commonly cites influences on adherence as side effects during MAD therapy, treatment efficacy, and the type of MAD employed [34].

Some limitations identified in this review include conducting a qualitative systematic review because only two studies met the inclusion criteria, heterogeneity in the methods of study evaluation, and lack of randomized clinical trials on the subject. These constraints can affect the synthesis, application, and generalizability of the reviewed information. Another potential challenge is the information bias stemming from our focus on studies published exclusively in English and involving adult populations. Another limitation is the diagnostic metrics used in the included studies. Both studies [15,21] adopted the Apnea–Hypopnea Index (AHI) as the primary outcome measure derived from full-night PSG, without reporting the Respiratory Disturbance Index (RDI). Respiratory effort-related arousals (RERAs) were excluded from the analysis. This may underestimate the total burden of sleep-disordered breathing events, particularly in patients with significant upper airway resistance without apneas or hypopneas. Future studies incorporating RDI could offer a more comprehensive assessment of the effectiveness of MAD therapies. Additionally, it should be noted that only one of the included studies [15] specified the version of the AASM scoring guidelines used (2007), whereas the other [21] did not. This lack of standardization in scoring criteria may introduce minor discrepancies in the definition of the apnea–hypopnea index (AHI), which could affect the comparability of results across studies. Nevertheless, our study is timely, particularly considering the increasing use of MADs. This study aimed to identify areas for improvement and advantages in using Neck CT and DISE to predict therapy outcomes in OSA, highlighting the importance of employing diverse tools for personalized and evidence-based therapeutic approaches.

The findings of this systematic review contribute to the broader field of sleep medicine by reinforcing the need for individualized diagnostic approaches in the management of OSA [15,21,30]. Given the phenotypic heterogeneity of OSA, no single diagnostic modality can reliably predict therapeutic success in mandibular MADs [2,15]. CT offers valuable anatomical insights into upper airway morphology [21], whereas DISE provides a dynamic and functional assessment that more closely reflects sleep-related airway behavior [15,26]. Within the broader framework of sleep medicine, these tools should be viewed as complementary elements of a multimodal evaluation strategy in line with the principles of precision medicine [26,30]. Their integration into clinical practice may facilitate the identification of patients most likely to benefit from non-CPAP therapies, such as MADs, particularly considering the well-documented challenges with long-term CPAP adherence [30–34]. Therefore, our findings underscore the importance of combining advanced diagnostic techniques with clinical, physiological, and patient-centered factors to optimize treatment outcomes in OSA [15,21,27].

## Conclusions

Our systematic review compared the predictive value of CT and DISE in evaluating the efficacy of MAD in adult individuals with OSA. Despite the limited number of studies included in our review, our findings provide valuable insights into the diagnostic utility of these modalities.

Our systematic review identified and analyzed two studies. The first study reported no significant differences in the ability of CT and DISE to predict the effectiveness of MAD therapy in OSA patients. Conversely, the second study suggested that only DISE was capable of accurately predicting MAD efficacy, indicating the potential superiority of DISE over CT.

The conflicting findings between the two studies underscore the complexity of OSA management and the challenges associated with predicting treatment outcomes using different diagnostic modalities. While CT offers detailed anatomical information regarding airway morphology, DISE provides dynamic visualization of upper airway collapse during sleep, which may better simulate real-life conditions and the MAD response.

### Future research

Future research should address these limitations by conducting well-designed prospective studies with larger sample sizes and standardized outcome measures. Comparative effectiveness research evaluating the predictive value of CT and DISE in larger cohorts of OSA patients undergoing MAD therapy is warranted to provide more robust evidence and inform clinical decision making.

### Handling missing data

No statistical imputation or estimation of missing data was performed in this review. For each included study, only reported data were extracted. If a specific data point was not available in the original article, this was recorded as “not reported” and was not included in the synthesis. Given the limited number of included studies (n = 2), and their prospective nature, no substantial missing data were observed that would affect the conclusions of this review.

## Supporting information

S1 File Minimal Data Set. (CSV)

S2 File All studies identified in the literature search. (CSV)

S3 File All study correspondence and exclusion reasons. (CSV)

S4 File PRISMA Checklist. (PDF)

## Abbreviations

AHI: Apnea-hypopnea index
AASM: American academy of sleep medicine
CBCT: Cone-beam computerized tomography
CCCp: Complete concentric palatal collapse
CFD: Computational fluid dynamics
DISE: Drug-induced sleep endoscopy
EMBASE: Excerpta medica dataBASE
ESS: Epworth Sleepiness Scale
ICSD-3: International classification of sleep disorders-3
IQR: Interquartile range
MAD: Mandibular advancement device
MCP: Maximum comfortable protrusive position
OSA: Obstructive sleep apnea
PSQI: Pittsburgh sleep quality index
QUADAS-2: Quality assessment of diagnostic accuracy studies-2
SaO2: Saturation of oxygen
T90: Time under 90% SaO2
UA: Upper airway.

## References

[1] Dinh-Thi-Dieu H, Vo-Thi-Kim A, Tran-Van H, Duong-Quy S. Efficacy and adherence of auto-CPAP therapy in patients with obstructive sleep apnea: a prospective study. Multidiscip Respir Med. 2020;15(1):468. doi: 10.4081/mrm.2020.468 32153777 PMC7037646

[2] Cebola P, Caroça C, Donato H, Campos A, Dias SS, Paço J, et al. Computed Tomography versus Sleep Endoscopy (DISE) to Predict the Effectiveness of Mandibular Advancement Devices in Adult Patients with Obstructive Sleep Apnea: A Protocol for Systematic Review. J Clin Med. 2023;12(19):6328. doi: 10.3390/jcm12196328 37834971 PMC10573249

[3] Palomo JM, Piccoli VD, Menezes LMD. Obstructive sleep apnea: a review for the orthodontist. Dental Press J Orthod. 2023;28(1):e23spe1. doi: 10.1590/2177-6709.28.1.e23spe1 37075419 PMC10108585

[4] Heinzer R, Vat S, Marques-Vidal P, Marti-Soler H, Andries D, Tobback N, et al. Prevalence of sleep-disordered breathing in the general population: the HypnoLaus study. Lancet Respir Med. 2015;3(4):310–8. doi: 10.1016/S2213-2600(15)00043-0 25682233 PMC4404207

[5] Kapur VK, Auckley DH, Chowdhuri S, Kuhlmann DC, Mehra R, Ramar K, et al. Clinical Practice Guideline for Diagnostic Testing for Adult Obstructive Sleep Apnea: An American Academy of Sleep Medicine Clinical Practice Guideline. J Clin Sleep Med. 2017;13(3):479–504. doi: 10.5664/jcsm.6506 28162150 PMC5337595

[6] Lv R, Liu X, Zhang Y, Dong N, Wang X, He Y, et al. Pathophysiological mechanisms and therapeutic approaches in obstructive sleep apnea syndrome. Signal Transduct Target Ther. 2023;8(1):218. doi: 10.1038/s41392-023-01496-3 37230968 PMC10211313

[7] Mediano O, González Mangado N, Montserrat JM, Alonso-Álvarez ML, Almendros I, Alonso-Fernández A, et al. International Consensus Document on Obstructive Sleep Apnea. Arch Bronconeumol. 2022;58(1):52–68. doi: 10.1016/j.arbres.2021.03.017 33875282

[8] Lévy P, Kohler M, McNicholas WT, Barbé F, McEvoy RD, Somers VK, et al. Obstructive sleep apnoea syndrome. Nat Rev Dis Primers. 2015;1:15015. doi: 10.1038/nrdp.2015.15 27188535

[9] Ottaviani G, Buja LM. Pathology of unexpected sudden cardiac death: Obstructive sleep apnea is part of the challenge. Cardiovasc Pathol. 2020;47:107221. doi: 10.1016/j.carpath.2020.107221 32371340

[10] Rodrigues AP, Pinto P, Nunes B, Bárbara C. Obstructive Sleep Apnea: Epidemiology and Portuguese patients profile. Rev Port Pneumol (2006). 2017;23(2):57–61. doi: 10.1016/j.rppnen.2017.01.002 28254339

[11] Attali V, Vecchierini M-F, Collet J-M, d’Ortho M-P, Goutorbe F, Kerbrat J-B, et al. Efficacy and tolerability of a custom-made Narval mandibular repositioning device for the treatment of obstructive sleep apnea: ORCADES study 2-year follow-up data. Sleep Med. 2019;63:64–74. doi: 10.1016/j.sleep.2019.04.021 31606651

[12] Cavaliere M, De Luca P, De Santis C, Scarpa A, Ralli M, Di Stadio A, et al. Drug-Induced Sleep Endoscopy (DISE) with Simulation Bite to Predict the Success of Oral Appliance Therapy in Treating Obstructive Sleep Apnea/Hypopnea Syndrome (OSAHS). Transl Med UniSa. 2020;23:58–62. doi: 10.37825/2239-9747.1011 33457325 PMC8370523

[13] Zhang P, Ye J, Pan C, Xian J, Sun N, Li J, et al. Comparison of drug-induced sleep endoscopy and upper airway computed tomography in obstructive sleep apnea patients. Eur Arch Otorhinolaryngol. 2014;271(10):2751–6. doi: 10.1007/s00405-014-3051-1 24748412

[14] Ramar K, Dort LC, Katz SG, Lettieri CJ, Harrod CG, Thomas SM, et al. Clinical Practice Guideline for the Treatment of Obstructive Sleep Apnea and Snoring with Oral Appliance Therapy: An Update for 2015. J Clin Sleep Med. 2015;11(7):773–827. doi: 10.5664/jcsm.4858 26094920 PMC4481062

[15] Van den Bossche K, Op de Beeck S, Dieltjens M, Verbruggen AE, Vroegop AV, Verbraecken JA, et al. Multimodal phenotypic labelling using drug-induced sleep endoscopy, awake nasendoscopy and computational fluid dynamics for the prediction of mandibular advancement device treatment outcome: a prospective study. J Sleep Res. 2022;31(6):e13673. doi: 10.1111/jsr.13673 35734809 PMC10078177

[16] Sakat MS, Sütbeyaz Y, Yüceler Z, Kantarci M, Kilic K, Kurt S. Cephalometric Measurements With Multislice Computed Tomography in Patients With Obstructive Sleep Apnea Syndrome. J Craniofac Surg. 2016;27(1):82–6. doi: 10.1097/SCS.0000000000002267 26745191

[17] Bharadwaj R, Ravikumar A, Krishnaswamy NR. Evaluation of craniofacial morphology in patients with obstructive sleep apnea using lateral cephalometry and dynamic MRI. Indian J Dent Res. 2011;22(6):739–48. doi: 10.4103/0970-9290.94566 22484864

[18] De Vito A, Carrasco Llatas M, Ravesloot MJ, et al. European position paper on drug-induced sleep endoscopy: 2017 update. Clin Otolaryngol. 2018;43(6):1541–52. 30133943 10.1111/coa.13213

[19] Vroegop AV, Vanderveken OM, Verbraecken JA. Drug-Induced Sleep Endoscopy: Evaluation of a Selection Tool for Treatment Modalities for Obstructive Sleep Apnea. Respiration. 2020;99(5):451–7. doi: 10.1159/000505584 32036366

[20] Moher D, Shamseer L, Clarke M, Ghersi D, Liberati A, Petticrew M, et al. Preferred reporting items for systematic review and meta-analysis protocols (PRISMA-P) 2015 statement. Syst Rev. 2015;4(1):1. doi: 10.1186/2046-4053-4-1 25554246 PMC4320440

[21] Saleh YAG, Fouda AM, El sayed Morsy N, Abdel-Khalek E, Saleh A. Effect of mandibular advancement device in the treatment of obstructive sleep apnea. The Egyptian Journal of Chest Diseases and Tuberculosis. 2022;71(3):343–52. doi: 10.4103/ecdt.ecdt_67_21

[22] Uniken Venema JAM, Bosschieter PFN, Hoekema A, Plooij JM, Lobbezoo F, de Vries N. Do dental parameters predict severity of obstructive sleep apnea and mandibular advancement device therapy outcomes? A pilot study. J Oral Rehabil. 2023;50(3):203–9. doi: 10.1111/joor.13392 36357333 PMC10107178

[23] Ristow O, Rückschloß T, Berger M, Grötz T, Kargus S, Krisam J, et al. Short- and long-term changes of the pharyngeal airway after surgical mandibular advancement in Class II patients-a three-dimensional retrospective study. J Craniomaxillofac Surg. 2018;46(1):56–62. doi: 10.1016/j.jcms.2017.10.022 29198376

[24] Vanderveken OM, Vroegop AV, van de Heyning PH, Braem MJ. Drug-induced sleep endoscopy completed with a simulation bite approach for the prediction of the outcome of treatment of obstructive sleep apnea with mandibular repositioning appliances. Operative Techniques in Otolaryngology-Head and Neck Surgery. 2011;22(2):175–82. doi: 10.1016/j.otot.2011.05.001

[25] Dieltjens M, Vanderveken O. Oral Appliances in Obstructive Sleep Apnea. Healthcare (Basel). 2019;7(4):141. doi: 10.3390/healthcare7040141 31717429 PMC6956298

[26] Vroegop AVMT, Vanderveken OM, Dieltjens M, Wouters K, Saldien V, Braem MJ, et al. Sleep endoscopy with simulation bite for prediction of oral appliance treatment outcome. J Sleep Res. 2013;22(3):348–55. doi: 10.1111/jsr.12008 23205856

[27] Van Gaver H, Op de Beeck S, Dieltjens M, De Backer J, Verbraecken J, De Backer WA, et al. Functional imaging improves patient selection for mandibular advancement device treatment outcome in sleep-disordered breathing: a prospective study. J Clin Sleep Med. 2022;18(3):739–50. doi: 10.5664/jcsm.9694 34608859 PMC8883076

[28] Chan ASL, Sutherland K, Schwab RJ, Zeng B, Petocz P, Lee RWW, et al. The effect of mandibular advancement on upper airway structure in obstructive sleep apnoea. Thorax. 2010;65(8):726–32. doi: 10.1136/thx.2009.131094 20685749

[29] Song B, Li Y, Sun J, Qi Y, Li P, Li Y, et al. Computational fluid dynamics simulation of changes in the morphology and airflow dynamics of the upper airways in OSAHS patients after treatment with oral appliances. PLoS One. 2019;14(11):e0219642. doi: 10.1371/journal.pone.0219642 31721777 PMC6853319

[30] Duong-Quy S, Nguyen-Huu H, Hoang-Chau-Bao D, Tran-Duc S, Nguyen-Thi-Hong L, Nguyen-Duy T, et al. Personalized Medicine and Obstructive Sleep Apnea. J Pers Med. 2022;12(12):2034. doi: 10.3390/jpm12122034 36556255 PMC9781564

[31] George JR, Chung S, Nielsen I, Goldberg AN, Miller A, Kezirian EJ. Comparison of drug-induced sleep endoscopy and lateral cephalometry in obstructive sleep apnea. Laryngoscope. 2012;122(11):2600–5. doi: 10.1002/lary.23561 23086863 PMC3484185

[32] Campos A, Cebola P, Dias SS, Pedro Pais J, Sousa S, Cardoso S, et al. Upper airway assessment in obstructive sleep apnea patients: can computed tomography with lateral cephalometry replace drug-induced sleep endoscopy (DISE)?. Acta Otorrinolaringol Esp (Engl Ed). 2023;74(5):290–7. doi: 10.1016/j.otoeng.2023.03.006 36990209

[33] Segù M, Cosi A, Santagostini A, Scribante A. Efficacy of a Trial Oral Appliance in OSAS Management: A New Protocol to Recognize Responder/Nonresponder Patients. Int J Dent. 2021;2021:8811700. doi: 10.1155/2021/8811700 34221017 PMC8225417

[34] van der Hoek LH, Rosenmöller BRAM, van de Rijt LJM, de Vries R, Aarab G, Lobbezoo F. Factors associated with treatment adherence to mandibular advancement devices: a scoping review. Sleep Breath. 2023;27(6):2527–44. doi: 10.1007/s11325-023-02862-9 37386300 PMC10656313

